# Paying Patients in Poor-Law Infirmaries

**Published:** 1921-05-14

**Authors:** 


### Paying Patients in Poor-Law Infirmaries.

Below we record the main points in this connection which were put forward by Mr. N. Bishop Harman when a deputation from the British Medical Association recently waited upon the Minister of Health. Technically these institutions are still part of the Poor Law, but in recent times their position has changed considerably. Patients can now be admitted who are not technically paupers. If for any reason a man is not able to get himself attended to when sick, he is "destitute" within that definition. This has now become the view of certain Boards of Guardians in this country, and thirteen such authorities have made it known that they are prepared to receive into their infirmaries persons who are able to pay for what they receive. The B.M.A. apparently welcome the development as enlarging the field for dealing with the sick, but they make suggestions for arrangements which they consider should be made before these schemes are officially acknowledged.

The first is that the local medical profession should necessarily be consulted, and the deputation urged that the Minister should issue this general recommendation to the authorities. Mr. Harman would like to see an *ad hoc* committee attached to the administration of all municipal hospitals, composed of representatives of the medical profession and of the municipal body. Their second point is that if these hospitals should be thrown open to a larger *clientèle*, patients who do not come by way of the relieving officer should only be admitted on the recommendation of the practitioner attending them. Some members of the deputation suggest an income limit, but the feeling is that the doctor's recommendation should overcome this necessity. The third point arises in connection with treatment in the hospitals. A certain number of beds might be relegated to wards recognised as those in which patients could be attended by the doctor of their choice. The medical superintendent would be in sole administrative control and the visiting doctor would only be allowed to come at certain definite hours. It is essential that a superintendent should have knowledge of the cases so that in emergency his help might be available, and the arrangement outlined, the B.M.A. consider, is nearly paralleled in the big voluntary hospitals. This "right of entry" should make the infirmaries approximate in some respects to the primary health centres of the Dawson Report. It is held that such a scheme would cost nothing to the State; indeed, the State might profit by it. It might be possible to capitalise the cost of the institution and include in the fee for maintenance a portion of the overhead charges for nursing. The Association's Hospital Committee have already drawn up a series of recommendations upon which after discussion with certain medical superintendents there is a fair basis of agreement. Upon the possibilities of such schemes articles have appeared in The Hospital on January 29, February 12 and 19.

